# The Effects of Technological Interventions on Social Participation of Community-Dwelling Older Adults with and without Dementia: A Systematic Review

**DOI:** 10.3390/jcm10112308

**Published:** 2021-05-25

**Authors:** Pascale Heins, Lizzy M. M. Boots, Wei Qi Koh, An Neven, Frans R. J. Verhey, Marjolein E. de Vugt

**Affiliations:** 1 Alzheimer Centrum Limburg, Department of Psychiatry and Neuropsychology, School for Mental Health and Neuroscience, Maastricht University, 6200 MD Maastricht, The Netherlands; p.heins@maastrichtuniversity.nl (P.H.); l.boots@maastrichtuniversity.nl (L.M.M.B.); f.verhey@maastrichtuniversity.nl (F.R.J.V.); 2 School of Nursing and Midwifery, National University of Ireland Galway, H91 TK33 Galway, Ireland; weiqi.koh@nuigalway.ie; 3 Transportation Research Institute (IMOB), UHasselt—Hasselt University, 3590 Diepenbeek, Belgium; an.neven@uhasselt.be

**Keywords:** social participation, technology, dementia, older adults, community

## Abstract

Social isolation in community-dwelling older adults with dementia is a growing health issue that can negatively affect health and well-being. To date, little attention has been paid to the role of technology in improving their social participation. This systematic review aims to provide a systematic overview of the effects of technological interventions that target social participation in community-dwelling older adults with and without dementia. The scientific databases Medline (PubMed), PsycINFO, CINAHL, Web of Science, and the Cochrane Library were systematically searched and independently screened by two reviewers. Results were synthesized narratively. The methodological quality of included studies was independently assessed by two reviewers. In total, 36 studies of varying methodological quality were identified. Most studies evaluated social networking technology and ICT training programs. Three studies focused on people with dementia. Quantitative findings showed limited effects on loneliness, social isolation, and social support. Nevertheless, several benefits related to social participation were reported qualitatively. Social interaction, face-to-face contact, and intergenerational engagement were suggested to be successful elements of technological interventions in improving the social participation of community-dwelling older adults. Rigorous studies with larger sample sizes are highly needed to evaluate the long-term effects of technology on the multidimensional concept of social participation.

## 1. Introduction

The world’s population is aging due to demographic changes. In 2020, 727 million people were aged 65 and over. According to the Department of Economic and Social Affairs of the United Nations, 1.5 billion people worldwide will be above this age by 2050 [1]. At the same time, the prevalence of dementia and other age-related neurodegenerative conditions is steadily increasing. Currently, there are 50 million people living with dementia worldwide. This number is expected to increase to 152 million by 2050 [2].

Social isolation is a growing health issue in the aged population [3,4]. It has been reported that more than 75 million adults in Europe experience social isolation [5]. In the United States, 24% of community-dwelling older adults were estimated to be socially isolated [6]. Recently, these numbers have increased rapidly across the globe due to the COVID-19 pandemic, especially among people with cognitive impairment [7]. In a recent Dutch study, more than half of the community-dwelling participants with cognitive decline reported not having any face-to-face contact with friends (52%) or family (57%) during the pandemic [7]. This kind of social isolation can negatively affect their mental and physical health [8,9], mortality [10], well-being, and quality of life [11]. Furthermore, poor social engagement is positively associated with an increased dementia risk [12,13,14]. Correspondingly, social relationships and participation in social activities can have a protective effect against cognitive decline and dementia [14,15]. However, community-dwelling people with dementia tend to experience difficulties participating in social situations, which might be the result of limited emotion perception [16], irritability, and fluctuating mood [17]. Moreover, due to the progressive deterioration of cognitive skills and the stigma associated with dementia, they are more likely to avoid social situations out of embarrassment or to even lose their interest in socializing in the community [18,19]. In combination with difficulties in spatial orienting, this avoiding behavior can result in limited participation in social activities, which subsequently can lead to social isolation and feelings of loneliness [19,20]. While social isolation refers to the objective lack of social connections, loneliness refers to the subjective feeling of lacking social connections [21].

There is a growing body of literature that has endorsed the urgent need for interventions targeting social participation in older adults. The definition of social participation, however, varies in the literature. In the renewed definition of health by Huber et al., social participation is one of the domains of social health and is described as “the ability to participate in social activities including work” [22] (p. 2). Whereas this definition focuses on the ability to socially participate, the definition by Levasseur et al. focuses on the element of social interaction [23]. According to them, social participation is defined as the “person’s involvement in activities that provide interaction with others in society or the community” [23] (p. 2148). Various studies in the field of gerontology have been using different concepts with comparable definitions, such as social connectedness [24] or social engagement [14]. Despite the indistinct use of these closely-related concepts, researchers agree on the potential benefits of psychosocial interventions in terms of (1) reducing social isolation or feelings of loneliness [3,8,25], (2) increasing well-being by fulfilling social needs [26,27], or (3) reducing mild cognitive impairment (MCI) and dementia risk [13]. Furthermore, several systematic reviews have highlighted the potential of technological interventions in enhancing the social participation of older adults [24,28,29,30]. Especially during the COVID-19 pandemic, the role of technological interventions has become crucial in preventing social isolation and loneliness [31].

Despite the vulnerability of people with dementia for social isolation, and their essential need for social contacts, very little attention has been paid to the effects of technological interventions that target social participation in this population [32,33]. To date, only one systematic review that explored the effects of ICT-based applications on the social participation of people with dementia could be identified [34]. The review includes different types of technology, such as electronic tagging technology, smart homes, and regular computers. The results of this systematic review indicate that technological solutions could facilitate and enhance social participation. Nevertheless, only two of the included studies had used a quantitative control group design, and the overall methodological quality of the included studies was—according to Pinto-Bruno et al.—poor [34]. In addition, the review covered literature published up to May 2016. It is likely that new technological interventions have been developed in the past five years, given the rising digital literacy among older adults [35]. As such, it is important to keep abreast of the developments. Since this review had a specific focus on people with dementia who live in residential care facilities, it is still unclear how technological interventions influence the social participation among older adults with dementia living in the community.

The present systematic review aims to provide a comprehensive overview of the effects of technological interventions that address social participation in community-dwelling older adults with dementia. In this paper, the definition of social participation by Levasseur et al. [23] will be used. Due to limited studies that directly target people with dementia, this systematic review included studies targeting older adults *with and without* dementia in order to provide a broader scope. Therefore, the following research questions were formulated: (1) what technological interventions have been studied that address the social participation of community-dwelling older adults with and without dementia, and (2) what are their effects and elements of success? It is anticipated that this research will contribute to a better understanding of technological interventions that have been studied and their role in enhancing social participation in community-dwelling older adults with and without dementia.

## 2. Materials and Methods

This systematic review was registered in the International Prospective Register of Systematic Reviews (PROSPERO) (registration number: CRD42020206654). It followed the procedures for systematic review reporting as stated by the Preferred Reporting Items for Systematic Reviews and Meta-Analyses (PRISMA) guidelines [36].

### 2.1. Search Strategy

In June 2020, the five electronic databases Medline (PubMed), PsycINFO, CINAHL, Web of Science, and the Cochrane Library were systematically searched. During the development of the search strategy, it was discovered that there are only limited studies referring to people with cognitive impairment. Consequently, the search was extended to studies that evaluated a technological intervention related to the social participation of community-dwelling older adults *with and without* cognitive impairment. The last search was conducted on 22 June 2020. In a later stage, citation tracing was used to identify additional studies from the reference lists of included studies and relevant systematic reviews [24,28,29,30,37].

The search strategy was based on the PICO model. It included synonyms of the following three categories: “older adults” (population), “technology” (intervention), and “social participation” (outcome). The search strategy used a combination of free text words with Medical Subject Headings (MeSH), Thesaurus terms, or CINAHL Subject Headings. It covered studies published between January 2000 and June 2020. The strategies for each of the electronic databases were developed and conducted by the first reviewer, discussed with the research team, and peer reviewed by an expert scientific information specialist of the Maastricht University Library. The full electronic search strategy conducted in Medline (PubMed) is displayed in Table 1.

### 2.2. Eligibility Criteria

Studies had to meet the following study characteristics to be included in the systematic review: (1) reported the effects of a technological intervention (no design restrictions imposed), (2) were aimed at community-dwelling older adults (defined as aged 55 and older) with or without cognitive impairment, (3) targeted social participation (defined as a “person’s involvement in activities that provide interaction with others in society or the community” [23] (p. 2148)) and/or social isolation, or reported effects related to social participation/social isolation, and (4) reported at least one outcome related to older adults with or without cognitive impairment. In order to ensure the accessibility of the systematic review findings within the international scientific community, only studies written in English were included. Furthermore, studies had to be published in 2000 or later to be included in the present review.

### 2.3. Study Selection

Two reviewers screened independently the titles and abstracts (P.H. and L.M.M.B.) and the full-texts (P.H. and W.Q.K.) of identified records, using a screening tool based on the eligibility criteria (see in Supplementary Materials). Discrepancies regarding the inclusion of full-text records were discussed with a third reviewer (M.E.d.V.) and resolved by consensus. If multiple reports of the same study were included in the systematic review, they were treated as a single study, after comparing for any discrepancies. The reference lists of included full-text records were screened by the first reviewer in order to possibly include additional studies. The records identified through citation tracing followed the same screening process as the records identified through the electronic search strategy.

### 2.4. Data Extraction

Data extraction of the included full-text records was performed by the first reviewer and checked by a second reviewer. Discrepancies regarding the extracted data were discussed between the two reviewers and resolved by consensus. The data extraction form (see in Supplementary Materials) was developed by the first reviewer and discussed with the research team. The form was pilot-tested on five randomly selected full-text records and subsequently adapted. When additional information or clarification regarding study data was required, the corresponding author of the study was contacted by mail.

Information relating to the general study characteristics was extracted, such as the country of data collection, the study aim, the study design, and the study population. To gain insight into the effects of technological interventions, information about the study outcomes (definition of outcomes, time points measured, outcome measures, and their validity) as well as the main findings/conclusions was extracted. Detailed information about the intervention was also extracted. This information included the aim, duration, timing, providers, setting, and theoretical basis of the intervention. In addition, text passages were extracted that possibly indicated factors explaining the success or failure of the technological intervention in influencing social participation.

### 2.5. Data Synthesis

Due to the heterogeneity of study designs, types of technologies, structure of interventions, and outcome measures of included studies, a quantitative synthesis of results was not appropriate. Therefore, a narrative synthesis was conducted to summarize the findings of included studies using descriptive tables and textual descriptions [38].

### 2.6. Quality Assessment

Two reviewers (P.H. and W.Q.K.) independently assessed the methodological quality of the included studies, discussed their individual ratings, and agreed on a final rating. Three different quality assessment tools were used, based on the study design of the included studies: quantitative, qualitative, or mixed methods. The kappa coefficients (κ) for the individual ratings of each assessment tool were calculated to determine the inter-rater agreement [39].

Given the variety in study design of included quantitative studies, the Effective Public Health Practice Project (EPHPP) tool was used to rate their methodological quality and to assess the risk of bias at the study level [40]. The tool consists of six component ratings, stimulating the reviewer to critically reflect whether: (1) study participants were representative of the study population, (2) randomization was used, (3) relevant confounders were described and controlled, (4) outcome assessor(s) and study participants were blinded, (5) data collection tools were shown to be valid and reliable, and (6) withdrawals and drop-outs were reported. The rating of each component (i.e., strong, moderate, or weak) was facilitated by a dictionary and led to an overall rating of the methodological quality (i.e., strong, moderate, or weak).

To appraise the methodological quality of included qualitative studies, a checklist based on the quality criteria synthesized by Walsh and Downe was used [41]. These quality criteria were chosen for their detailed description and their coverage of the concept of trustworthiness in qualitative research as defined by Lincoln and Guba [42]. Within the checklist, each of the 12 criteria was rated (i.e., criterion met, criterion partly met, or criterion unmet), covering the following categories: scope and purpose, design, sampling strategy, analysis, interpretation, reflexivity, ethical dimensions, and relevance and transferability. In a next step, points were awarded for each rated criterion (i.e., criterion met = 1 point, criterion partly met = 0.5 points, criterion unmet = 0 points). By adding up the points, an overall rating of the methodological quality with a maximum of 12 points was determined.

Studies in this systematic review were considered as mixed methods studies as long as a combination of qualitative and quantitative data collection and analysis procedures was described. Based on the recommendations of Heyvaert et al. [43], the methodological quality of mixed methods studies was appraised using the EPHPP tool for the quantitative part of the study, the quality criteria by Walsh and Downe for the qualitative part of the study, and the mixed methods criteria by Creswell and Plano Clark to evaluate the integration of both parts [44]. The latter consists of four criteria that evaluate whether: (1) the collection and analysis of quantitative and qualitative data were rigorous, (2) the integration of both data types was included in the results section, (3) the mixed methods research design was chosen logically, and (4) the mixed methods design was surrounded by theory and philosophy. Each of the four criteria was rated (i.e., criterion met, criterion partly met, or criterion met). Points were assigned for each rating (i.e., criterion met = 1 point, criterion partly met = 0.5 points, criterion unmet = 0 points), leading to an overall rating of the methodological quality with a maximum of 4 points.

## 3. Results

### 3.1. Study Selection

**Figure 1:**
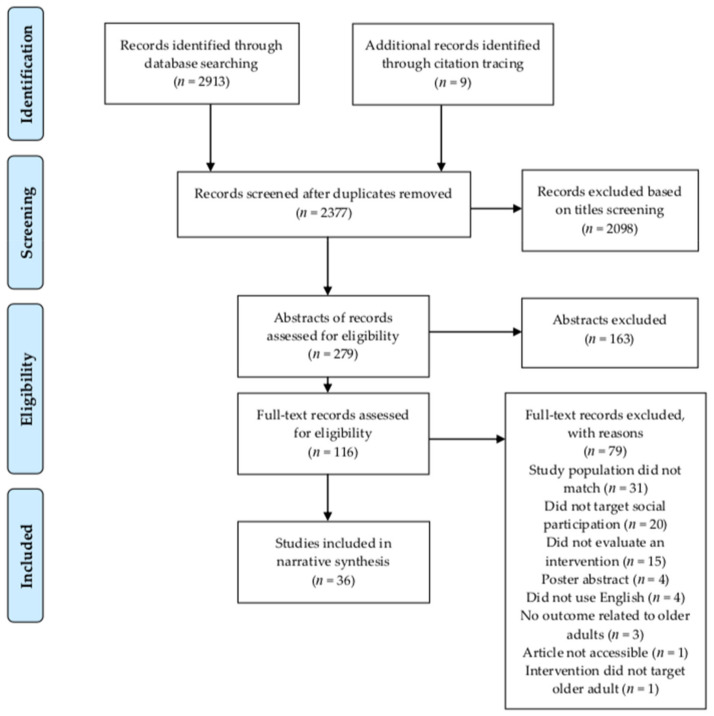
Study selection flowchart based on PRISMA guidelines.

Figure 1 visualizes the study selection process based on the PRISMA guidelines [36]. The database search yielded 2913 records. After title and abstract screening, 79 of the 107 screened full-text records were excluded based on various reasons (see Figure 1). Next to the database search, 9 additional full-text records were identified through citation tracing. Two reports of the same study were identified and treated as one single study. As such, a total of 37 reports, covering 36 studies, met all of the inclusion criteria and were subsequently included in this systematic review.

### 3.2. Characteristics of Examined Studies

#### 3.2.1. General Study Characteristics

Studies were published between 2005 and 2020. While 12 studies were published before or in the year 2015 [45,46,47,48,49,50,51,52,53,54,55,56], 24 studies were published after the year 2015 [57,58,59,60,61,62,63,64,65,66,67,68,69,70,71,72,73,74,75,76,77,78,79,80]. Studies were conducted in 11 different countries, with most of them being conducted in the USA $\left(n=17\right)$. The majority of the included studies used a qualitative study design $\left(n=14\right)$ [45,47,48,49,57,59,60,61,62,66,68,69,75,76]. Of the remaining studies, 12 were of quantitative nature [46,52,55,56,63,65,67,71,73,74,78,80], and 10 studies were considered as mixed methods studies [50,51,53,54,58,64,70,72,77,79].

#### 3.2.2. Study Population Characteristics

Sample sizes $\left(M=53.86,\;SD=72.23\right)$ ranged from 5 [57] to 300 [63], with a majority of the participants being female. While most studies targeted older adults in general (with and without cognitive impairment), one third of the studies excluded older adults with cognitive impairment. Only three of the included studies evaluated the effect of technology on older adults with dementia: two qualitative studies [62,76] and one quantitative [78]. Next to that, one study focused on low-income older adults [69]. Several studies additionally evaluated the perceptions of other stakeholders: informal caregivers [48,76,78], family members [49,79], friends [79], volunteers [57,68] service coordinators [57], and young adult mentors [59].

#### 3.2.3. Intervention Characteristics

Studies were heterogenous in terms of intervention characteristics. A third of the included studies $\left(n=12\right)$ focused on communication and social networking technology [45,46,47,49,51,54,57,61,66,68,73,79], and 10 evaluated ICT training programs [55,56,58,59,60,64,69,70,72,74]. Interestingly, the more recently published studies that evaluated these ICT training programs incorporated the concept of “reverse mentoring” [59,70,72]. Within this concept, young adults or students act as mentors and training instructors for older adult participants. Few studies addressed mobile applications $\left(n=4\right)$ [65,67,78,80] and gaming technology $\left(n=4\right)$ [52,62,75,76]. The remaining studies examined the effect of: activity-based musical engagement with iPads [50], a tablet-based language training program [77], the provision of Internet access [53], technology-assisted self-monitoring of physical activity [71], a Personal Reminder Information and Social Management (PRISM) system [63], and telecare [48].

In addition to the various types of technological interventions, the modality and aim of technological interventions varied as well. While 22 technologies included some kind of face-to-face contact, 14 technologies were fully virtual in nature [46,48,49,51,53,54,57,61,63,65,66,68,75,79]. Only 2 of the 36 included studies highlighted a explicit primary intervention aim to increase social participation [64,67]. Nonetheless, numerous studies mentioned addressing other social outcomes, such as social isolation and loneliness [47,51,54,56,57,65,70,71,72,74,75,79]. Although some of the remaining studies $\left(n=7\right)$ stated non-social intervention aims, such as improving cognitive function or increasing comfort with technology, more than half of them $\left(n=15\right)$ did not explicitly state an intervention aim at all [45,49,50,52,53,55,58,59,60,61,62,63,66,68,77].

### 3.3. Outcomes Related to Social Participation

Different variables were used to assess the effects of technological interventions on social participation outcomes. Loneliness was the most frequently measured psychosocial outcome identified in quantitative and mixed methods studies [46,51,52,54,55,56,63,65,67,70,72,73,74,77,80], followed by perceived social support [56,63,73,74,80] and social isolation [50,63,70,74]. Interestingly, the variables of loneliness, social isolation, and (perceived) social support were not measured coherently. While most of the studies measured loneliness as a distinct variable, Lee and Kim measured loneliness together with perceived social support as part of the concept of social isolation [70]. Slegers, van Boxtel, and Jolles assessed loneliness combined with the frequency and nature of the participants’ social network to evaluate the concept “social well-being” [55].

Less frequently measured outcomes included social network size [63], social integration [73], social connectedness [53,79], and social interaction [71,78]. Only one study explicitly stated social participation as quantitative outcome of interest [64]. Emas et al. used self-developed scales to assess social participation, defined in their study as the participants’ skill ability and confidence level in iPhone/iPad use. While the majority of included studies assessed the psychosocial outcomes at pretest and posttest, four studies assessed the outcomes one to three months after the intervention [51,56,72,74].

### 3.4. Quality Assessment

There was substantial agreement (κ = 0.699) between the two reviewers for the quality appraisal of quantitative studies before consensus was reached on the final rating [39]. Three quantitative studies were rated as strong [55,63,78]; three were rated as moderate [71,73,80]; and six were rated as weak [46,52,56,65,67,74]. From the data in Table 2, it is apparent that the quality of most study designs was strong. Eight of the quantitative studies were at first considered as Randomized Controlled Trials (RCTs) [46,52,55,56,63,71,78,80]. However, three studies did not describe the randomization procedure in their study methodology [46,52,56]. According to the EPHPP guidelines, these studies were classified as Clinical Controlled Trials (CCTs). One additional study was classified as a CCT because the randomized allocation of study participants conflicted with participants’ availability. The remaining quantitative studies were classified as single-group cohort studies [65,67,74].

Several study aspects led to a lower methodological quality rating among quantitative studies. Overall, these studies were subjected to a high chance of selection bias. Only one study had a likely representative study sample [55]. Moreover, most included studies did not specify in most cases the percentage of selected individuals that agreed to participate in the study. In addition, several studies (n= 6) did not mention about the blinding of outcome assessors.

For the methodological quality ratings of qualitative studies, a kappa coefficient (κ) of 0.415 was achieved, which indicated a moderate agreement between the two reviewers before consensus was reached on the final rating [39]. Table 3 shows a detailed overview of the individual criteria ratings and total ratings assigned for the methodological quality of included qualitative studies. The final ratings ranged from 5.5 [60] to 9 [66,76] out of 12. In general, included studies clearly stated their research aims or questions and were thoroughly contextualized by existing literature. However, a majority of the qualitative studies did not discuss the relationship between researcher and study participants nor the potential influence of researchers on the research process. Some studies did not provide a justification for the chosen sampling methods and the analytic approach used.

The kappa coefficient (κ) for the quality assessment of mixed methods studies equaled 0.617, which indicated a substantial agreement between the two reviewers before consensus was reached on the final rating [39]. The methodological quality ratings of mixed methods studies are displayed in Table 4. What stands out in the table is that both the quantitative and mixed methods final ratings indicate a weak methodological quality. None of the studies framed the mixed methods procedures within theory and philosophy. Moreover, most mixed methods studies did not integrate the qualitative and quantitative data strands. The qualitative final ratings ranged from 4 to 9 out of 12. Only a limited number of mixed methods studies discussed choices and procedures concerning qualitative sampling, data collection, and analysis in detail.

### 3.5. Effects of Interventions on Social Participation

#### 3.5.1. Quantitative Findings

Table 5 synthesizes the findings of each included quantitative study, listed in descending order based on their methodological quality. Very few of the quantitative studies found statistically significant effects on social participation outcomes. Only one quantitative study could be identified that addressed exclusively community-dwelling older adults with dementia [78].

Three quantitative studies with a strong methodological quality rating did not find significant group differences at post-intervention follow-up. In the study by Slegers, van Boxtel, and Jolles [55], no significant intervention effect on social well-being or any other dimension of well-being could be identified. In contrast, in the study by Czaja et al. [63], significant changes between the two groups in the domains of loneliness and perceived social support were identified at mid-term follow-up. In addition, a decrease in social isolation could be found. Similarly, in the RCT by Yu et al. [78], outcomes related to social participation significantly improved at mid-term follow-up. While there were no significant differences between the three group in the primary outcome “mood”, there was a significant higher social interaction in the intervention group compared with the control and comparison group at 6 weeks. However, the significant group differences at mid-term follow-up reported in these two latter studies could not be maintained at post-intervention follow-up [63,78].

Six of the remaining nine quantitative studies failed to demonstrate statistically significant intervention effects on social participation outcomes in the intervention group [46,56,67,71,73,74]. It has to be noted that a majority of these studies were feasibility trials [46,67,71,74]. Two technological interventions did not focus on outcomes related to social participation as a primary aim [71,73]. Both studies found statistically significant effects on their primary outcomes of interest: physical activity [71] and cognitive function [73]. Of the three quantitative studies that reported statistically significant changes in social participation outcomes, two reported a significant decrease in loneliness among older adults [52,65]. Moreover, one study found a significant interaction effect for informational and tangible support [80].

#### 3.5.2. Qualitative Findings

Synthesized information about the intervention, design, and main findings of included qualitative studies can be found in Table 6, sorted in descending order based on the studies’ quality assessment rating. Qualitative studies reported various benefits of technological interventions on the social participation of older adults. These benefits included: (1) maintenance or development of social relationships or connections [47,48,57,59,66,68,69], (2) improvements in social connectedness [49,60], (3) decrease in loneliness [45,57], (4) companionship and social interaction [61], and (5) improvements in communication [68]. In addition, study participants reported benefits of technological interventions in terms of life satisfaction [60], ICT skills [47], confidence in the use of technology [59], or physical activity [61]. Only two qualitative studies focused on community-dwelling older adults with dementia [62,76].

#### 3.5.3. Mixed Methods Findings

A summary of findings from mixed methods studies is detailed in Table 7. Less than half of the studies revealed significant intervention effects on social participation outcomes. One study found a significant increase in social participation among older adult participants [64]. In this study, social participation was measured as the participants’ skills ability and confidence level in several ICT-related tasks. Furthermore, three studies reported a statistically significant decrease in loneliness [54,70,72]. In addition to this, Lee and Kim [70] also found a decrease in total social isolation. The remaining six mixed methods studies did not find any statistically significant intervention effects on social participation outcomes [50,51,53,58,77,79]. All of them were carried out with small sample sizes and five of them were described as pilot studies [50,51,53,58,77,79].

Based on qualitative findings, study participants reported overall positive effects on social participation, such as enhanced social connectedness [53,72] and enhanced ICT skills that facilitated the communication with loved ones [64]. While one study found that participants in a group intervention developed social cohesion and group identity [50], two other studies found that participants in group intervention programs had a lack of group cohesion and social ties [51,77].

## 4. Discussion

This systematic review is, to our knowledge, the first to assess the potential of technological interventions in enhancing the social participation of community-dwelling older adults, including people with dementia. The primary aim of this review was to provide a comprehensive overview of technology that has been studied and used to address social participation in this study population. In total, 36 studies were included. A variety of technological interventions were identified, most of them being communication and social networking technology and ICT training programs. These findings correspond with findings from other systematic reviews that looked at the use of technology to address social isolation among older adults in general [28,29,30]. Baker et al. [30], however, identified pet robots as another technology-based intervention. As pet robot evaluations are mostly conducted in institutional care settings, it is not surprising that none of the included studies evaluated pet robots in community settings [81].

Based on the recency of the studies, we note an apparent shift away from computer-based interventions to more tablet- and smartphone-based interventions. This may be due to a greater ease of access to tablets and smartphones as compared to computer-based solutions since the former options are usually incorporated into one’s everyday life. Despite the fact that tablet- and smartphone-based technologies are portable and non-location bound, most studies that used these technologies conducted the interventions in participants’ homes. However, prior studies have shown that the engagement in community-based out-of-home activities can contribute to the social participation of older adults [82,83]. Considering this, it is surprising that only one study used technology to facilitate social participation in the community, by encouraging older adults via the mobile application “GezelschApp” to physically engage in community-based activities with others [67]. This finding may be explained by the fact that most studies did not have an explicit focus on improving social participation. Instead, their focus was on reducing the negative consequences of social isolation. This highlights the empirical consequences of having a poorly defined concept of social participation.

### 4.1. Effects of Interventions on Social Participation

Overall, quantitative and mixed methods studies showed that the use of technological interventions had limited effects on loneliness, social isolation, and social support. This may be attributed to the fact that many studies were pilot studies with small sample sizes. Nevertheless, qualitative findings were able to identify various benefits, such as the development of social connections and the improvement of social connectedness. As expected, only limited studies addressing people with dementia could be identified $\left(n=3\right)$ [62,76,78]. Due to the heterogeneity of study and intervention characteristics and the limited number of studies that targeted older adults with dementia, no subgroup analyses could be carried out to evaluate whether the effects may differ between people with and without dementia. Based on the body of evidence that has been synthesized narratively, no conclusion can be drawn regarding the effectiveness of technological interventions for people with dementia. Consequently, the present findings suggest that technological interventions may have the potential to alleviate loneliness and enhance social support in cognitively healthy older adults. As the effects on loneliness and social support were inconsistent, findings have to be interpreted with caution.

Such inconsistent findings were also reported in other systematic reviews in similar fields. Although the effects of ICT on loneliness were inconclusive in the review by Chen and Schulz [29], ICT was found to positively affect social connectedness, social isolation, and social support. A majority of the studies included in the review by Khosravi et al. [30] found positive effects in reducing loneliness or social isolation. These inconsistent study findings may be due to the use of different inclusion criteria. Khosravi et al. [30] for example included exclusively quantitative study findings related to a younger age group (older adults aged 50+) living in institutional care or community settings. In contrast, the present systematic review focused on various research designs including older adults aged 55 years or older living in a community setting. Regarding the effectiveness of technology on the social participation of people with dementia, other systematic reviews also failed to identify a high number of studies with rigorous study designs [34,37].

Findings of the present systematic review suggest that social interaction, face-to-face contact, and intergenerational engagement may be successful elements of technological interventions in enhancing the social participation in community-dwelling older adults. All seven studies that found a statistically significant intervention effect included a social interaction element [52,54,64,65,70,72,80]. This finding is not surprising, as social participation in the present review was defined as a “person’s involvement in activities that provide interaction with others in society or the community” [23] (p. 2148). Moreover, it is noteworthy that five of the seven studies had the element of a face-to-face contact [52,64,70,72,80]. Interestingly, three studies that demonstrated significant changes contained the element of intergenerational engagement between older adult study participants and younger adult students [52,70,72]. Gardiner, Geldenhuys, and Gott [84] looked at elements that should be included in interventions to successfully address social isolation and/or loneliness in the aged population. Three categories were identified: adaptability, productive engagement, and the use of a community development approach. Engagement within a group to socialize and build social connections was part of the productive engagement category. Furthermore, ten Bruggencate, Luijkx, and Sturm [26] argued that an intervention is successful in enhancing the social well-being of older adults when targeting their social needs. Interestingly, all of the three facilitators of social participation identified in the present systematic review could be seen as a way to fulfill the social need of social connections and connectedness [26,33]. As a consequence, a question arises around whether the technology is an intermediary to fulfill this social need rather than the technology producing the effect.

A major finding from our study was the inconsistent use of terms and concepts related to social participation. Social participation is a multidimensional concept that comprises the dimensions of social connections, volunteering, and engaging with others in activities for personal enjoyment [23,85]. However, most of the included studies in the present systematic review measured only one specific dimension of social participation: the dimension of social connections. Likewise, other systematic reviews that looked into interventions for social isolation found that a majority of studies mainly assessed outcomes related to this dimension, such as loneliness and social support [29,86]. While social isolation and loneliness are distinct concepts, they are sometimes used interchangeably by researchers. As social isolation refers to an actual lack of social connections [21], the facilitation of social participation can be considered equal to the decrease of social isolation [28]. More importantly, an actual improvement and enrichment of social connections does not necessarily mean a decrease in loneliness, defined as the subjective feeling of lacking social connections [21]. Given these points, the comparability of study findings is limited.

### 4.2. Methodological Quality of Included Studies

Overall, the methodological quality of studies was inconsistent across study designs. It has to be noted that the EPHPP component ratings “blinding” and “confounders” of several studies were difficult to rate due to the lack of a control group. As the EPHPP dictionary did not provide recommendations on how this issue should be addressed, the first author standardized the process by rating these component ratings as weak. In addition, a high selection bias could be observed across quantitative and mixed methods studies because study participants were mainly self-referred. This selection bias might have implications for the findings of the present systematic review. As mentioned before, limited quantitative evidence was found regarding the effectiveness of technological interventions on social participation. An explanation might be that older adults who are contacted and willing to participate in a study are not socially isolated. This explanation can be supported by the included studies that reported a lack of study participants with a high degree of social isolation at baseline [50,73,74,75]. In addition, the study participants’ digital literacy, attitudes toward technology, and investment in learning how to use a certain technology may have led to biased study findings. This issue was not covered by the used quality assessment tools. However, some studies addressed this issue by evaluating attitudes toward technology, use of technology, or digital literacy prior to the intervention. Other studies addressed this issue by providing training sessions on how to use the technology or by including study participants based on their digital literacy.

### 4.3. Strengths and Limitations

The strength of this study lies in the systematicity of our approach. The systematic search strategy that combined free text words with Medical Subject Headings (MeSH), Thesaurus terms, and CINAHL Subject Headings ensured the inclusion of relevant studies from various scientific databases. Moreover, the continuous involvement of two independent researchers throughout the entire review process enhanced the rigor of the screening and quality appraisal process. Using three different quality assessment tools furthermore enabled a thorough quality appraisal of the included studies, regardless of their design.

A number of potential limitations need to be considered. Firstly, a publication bias might have occurred, as this review was restricted to published results. Furthermore, the search was limited to studies written in English and published in peer-reviewed journals. Hence, research findings published in other sources, such as trade journals, were excluded. As such, potentially relevant publications may have been missed. Nevertheless, measures were taken to ensure the comprehensiveness of our search: the use of citation tracing, the use of different search terms related to the concept of social participation, and the inclusion of database-specific subject headings. Despite this, the search terms used in this systematic review may not cover all relevant studies since researchers use different terms to refer to the multidimensional concept of social participation. Secondly, included studies might be falsely considered as mixed methods studies, even when the respective study authors did not intentionally use a mixed methods design. Consequently, the methodological quality of those studies may have been rated inappropriately. Lastly, this systematic review included studies with diverse research designs and inconsistent methodological quality. The generalizability of the review findings may as a result be limited. However, the diversity of study designs broadened the scope of this review, identifying various technological interventions that target the social participation of community-dwelling older adults.

### 4.4. Implications for Practice and Future Research Directions

The present findings have implications for targeting the growing health issue of social isolation in the aged population. Due to the COVID-19 pandemic, social interaction occurred mainly digitally during the past year. As a consequence, older adults living in the community were not only socially excluded from society but were also digitally excluded from society [87,88]. Based on the present review findings, technology could play a crucial role in addressing these consequences of COVID-19 in community-dwelling older adults by providing opportunities to maintain social connections and to socially participate in the community. Policy makers should therefore prioritize the need of older adults to socially participate in the community and provide technological services to address this need. To ensure that these services can successfully address social participation, they should incorporate a social interaction element and provide possibilities to engage in out-of-home activities. Moreover, designers of technological interventions that target social participation might incorporate social interaction elements into the intervention.

As new technological solutions emerge every year and the number of older adults experiencing social isolation grows rapidly, future studies need to be carried out to evaluate the impact of emerging technologies on social participation outcomes. To capture the complex nature of social participation, it would be of value for studies to collect both qualitative and quantitative data. Furthermore, there is a need for a consistent terminology. Researchers should consider identifying and clearly defining the concept of interest in their studies. Due to a lack of an outcome measure that covers diverse social participation dimensions, studies should combine several validated outcome measures to assess social participation. Future studies should consider developing and validating an outcome assessment that covers all dimensions of social participation. Different health care professionals, such as occupational therapists, could use the tool to tailor their intervention to the individual needs of older adults living in the community. As the purpose of social participation is to facilitate the involvement in activities in the community [23], more studies should look into using technology to facilitate the participation in out-of-home social activities. In order to evaluate the long-term effects of technological interventions on social participation, studies with larger sample sizes that focus on older adults with a high degree of social isolation are needed. In addition, researchers not only should look into the effects of technological interventions, but also should explore whether these effects are mediated rather than produced by the technology.

In this systematic review, a lack of studies that address community-dwelling older adults with dementia was identified. Therefore, an important aspect to explore in future studies is the potential of technological interventions to reduce the social isolation of this study population. Moreover, it is likely that factors other than the ones identified in this review play a role in facilitating the social participation of people with dementia. According to Dröes et al. [89], personal and disease-related factors, social environment factors, and physical environment factors can impede or enhance their social participation. Similarly, the findings of Gaber et al. [90] suggest that contextual factors, such as characteristics of the physical and social environment, need to be considered when enhancing the social participation of people living with dementia. Further research should focus on identifying factors of technological interventions that facilitate social participation in older adults and, particularly, in older adults with dementia.

## 5. Conclusions

Technological interventions have shown the potential to alleviate loneliness and social isolation and to enhance social support. In particular, technological interventions that contain the elements of social interaction, face-to-face contact, or social engagement seemed to be most effective. This review is a starting point for future research regarding the use of technology to facilitate social participation among older adults and to thereby reduce their (risk of) social isolation, especially in the context of dementia.

## Figures and Tables

**Table 1:** Full search strategy of electronic database Medline (PubMed).

Categories	Search Terms
#1 Population synonyms	Middle Aged[MeSH] OR middle aged[title/abstract] OR Aged[MeSH] OR aged[title/abstract] OR elderly[title/abstract] OR older adults[title/abstract]
#2 Intervention synonyms	Technology[title/abstract] OR technological[title/abstract] OR technologies[title/abstract]
#3 Outcome synonyms	Community Participation[MeSH] OR community participation[title/abstract] OR Social Participation[MeSH] OR social participation[title/abstract] OR Interpersonal Relations[MeSH] OR interpersonal relations[title/abstract] OR Social Isolation[MeSH] OR social isolation[title/abstract] OR social health[title/abstract] OR social activity[title/abstract] OR social activities[title/abstract] OR social interaction[title/abstract]
Limiters	Results by year: from 2000–2020
#4 Combination of categories	#1 AND #2 AND #3

**Table 2:** Quality appraisal of quantitative studies (EPHPP) $\left(n=12\right)$.

Study	Selection Bias	Study Design	Confounders	Blinding	Data Collection Methods	Withdrawals and Drop-Outs	Global Rating
[78]	2	1	1	2	1	1	1
[55]	2	1	1	3	1	1	1
[63]	2	1	1	2	1	2	1
[71]	3	1	1	2	1	1	2
[73]	3	1	1	2	1	1	2
[80]	3	1	1	2	1	1	2
[46]	2	1	3	3	1	1	3
[56]	3	1	1	3	3	2	3
[52]	3	1	3	3	3	1	3
[67]	3	2	3	3	3	1	3
[74]	3	2	3	3	1	3	3
[65]	3	3	3	3	3	1	3

Notes: 1 = strong, 2 = moderate, 3 = weak. Studies are named according to their reference number within this systematic review.

**Table 3:** Quality appraisal of qualitative studies (Walsh and Downe criteria) $\left(n=14\right)$.

Criteria	[66]	[76]	[61]	[45]	[47]	[48]	[59]	[68]	[69]	[75]	[62]	[57]	[49]	[60]
Clear statement of, and rationale for, research question/aims/purposes	+	+	+	+	+	+	+	+	+	+	+	±	±	±
Study thoroughly contextualized by existing literature	+	+	±	+	+	±	+	+	+	+	+	+	+	+
Method/design apparent and consistent with research intent	+	±	±	±	+	±	±	–	±	–	±	±	±	±
Data collection strategy apparent and appropriate	±	+	+	±	+	+	±	±	±	±	±	±	±	±
Sample and sampling method appropriate	±	±	±	±	±	±	±	+	±	±	±	–	±	±
Analytic approach appropriate	±	±	±	±	–	±	±	±	±	±	±	±	–	±
Context described and taken account of in interpretation	+	+	±	±	±	±	±	+	+	+	±	±	±	±
Clear audit trail given	±	+	+	±	±	±	±	±	±	±	±	±	±	–
Data used to support interpretation	+	±	+	±	±	+	+	+	±	+	+	+	±	±
Researcher reflexivity demonstrated	–	±	–	±	+	±	–	–	–	–	–	–	±	–
Demonstration of sensitivity to ethical concerns	+	±	±	±	–	±	±	–	±	±	±	±	±	±
Relevance and transferability evident	+	+	+	+	±	±	+	+	+	+	±	+	±	±
Total score	9	9	8	7.5	7.5	7.5	7.5	7.5	7.5	7.5	7	6.5	6	5.5

Notes: +, criterion met (=1 point), ±, criterion partly met (=0.5 points), –, criterion unmet (=0 points). By adding up the points, a total score of the methodological quality with a maximum of 12 points was determined. Studies are named according to their reference number within this systematic review.

**Table 4:** Quality appraisal of mixed methods studies (EPHPP, Walsh and Downe criteria, and Creswell and Plano Clark criteria) $\left(n=10\right)$.

	Assessment Tools	Criteria												
	**EPHPP**	**Selection Bias**		**Study Design**					**Confounders**	**Blinding**	**Data Collection Methods**		**Withdrawals and Drop-Outs**	**Final Rating**
[51]		3		1					1	3	1		2	3
[77]		3		2					3	3	1		3	3
[70]		2		2					3	3	3		3	3
[72]		3		3					3	3	3		3	3
[50]		3		1					3	3	1		1	3
[79]		3		2					3	3	3		1	3
[58]		2		2					3	3	3		3	3
[53]		3		2					3	3	1		2	3
[54]		3		2					3	3	3		3	3
[64]		3		2					3	3	3		3	3
	**Qualitative** **criteria**	**Study purpose**	**Study scope**	**Study Design**	**Data collection**	**Sampling strategy**	**Analysis**	**Study context**	**Audit trail**	**Data to support interpretation**	**Reflexivity**	**Ethical dimensions**	**Transferability**	**Final rating**
[51]		+	±	±	+	±	±	+	+	±	±	+	+	9
[77]		+	±	±	+	±	±	±	±	+	–	±	±	7
[70]		±	±	–	±	±	±	+	±	+	–	±	+	6.5
[72]		+	+	±	±	±	±	±	±	–	–	±	±	6
[50]		+	±	–	±	±	±	–	±	±	±	±	±	5.5
[79]		+	+	±	±	±	–	±	±	±	–	–	±	5.5
[58]		+	±	–	±	±	±	±	–	±	–	±	±	5
[53]		+	+	±	±	–	–	–	–	±	–	±	±	4.5
[54]		+	+	–	±	±	–	±	–	–	–	±	±	4.5
[64]		+	±	–	±	±	–	±	–	±	–	–	±	4
	**Mixed methods criteria**	**Frames the procedures within theory and philosophy**					**Organizes the procedures into specific research designs**			**Collects and analyses both qualitative and quantitative data rigorously**		**Intentionally integrates the two data strands**		**Final rating**
[51]		–					±			±		–		1
[77]		–					±			+		–		1.5
[70]		–					±			±		–		1
[72]		–					±			±		–		1
[50]		–					±			±		±		1.5
[79]		–					–			±		±		1
[58]		–					±			±		–		1
[53]		–					±			±		±		1.5
[54]		–					±			±		–		1
[64]		–					±			±		–		1

Notes: EPHPP: 1 = strong, 2 = moderate, 3 = weak; Qualitative criteria: +, criterion met (=1point); ±, criterion partly met (=0.5 points); –, criterion unmet (=0 points). By adding up the points, a total score of the methodological quality with a maximum of 12 points was determined; Mixed methods criteria: +, criterion met (=1point); ±, criterion partly met (=0.5 points); –, criterion unmet (=0 points). By adding up the points, a total score of the methodological quality with a maximum of 4 points was determined. Studies are named according to their reference number within this systematic review.

**Table 5:** Design, methods, and findings related to social participation reported from included quantitative studies.

Authors (Year), Country	Study Design ^1^	Experimental Intervention	Control or Comparison Intervention	Setting	Participants (n = Sample Size)	Outcomes Related to Social Participation	Outcome Measures Related to Social Participation	Findings Related to Social Participation
Yu et al. (2019) [78], USA	RCT ^2^	Mobile reminiscing therapy app “Memory Matters”: one-on-one 30 min sessions with an interventionist (2×/week) for 6 weeks followed by independent use for 6 weeks	Comparison: group 30 min sessions with an interventionist (2×/week) followed by group 30 min sessions with an activity director. Control: waitlist.	Older adults’ residence	Older adults with dementia $\left(n=80\right)\;$ and caregivers	Social Interaction (pretest, 6 weeks, posttest)	Pleasant Events Schedule-AD (PES-AD short version)	6 weeks: significant higher social interaction of the individual MM group vs. the comparison $\left(t=2.38,\;p=0.017\right)$ and the control group $\left(t=2.84,\;p=0.005\right)$. 12 weeks: not maintained.
Slegers, van Boxtel, and Jolles (2008) [55], the Netherlands	CCT ^3^	Computer training program: 3 × 4 hr training sessions for 2 weeks, independent use of the computer combined with assignments (1×/2 weeks in the first 4 months, 1×/month for the last 8 months)	Comparison: 3 × 4 h training sessions for 2 weeks, followed by independent computer use. Control: No intervention.	Home-based (setting of training sessions not mentioned)	Older adults without cognitive impairments $\left(n=236\right)$	Social well-being (pretest, 4 months, posttest)	De Jong Gierveld Loneliness Scale, self-reported nature, and frequency of social network	No significant positive (or negative) intervention effect on social well-being.
Czaja et al. (2018) [63], USA	CCT	Personal Reminder Information and Social Management (PRISM) system: use of the computer system for 12 months	Comparison: use of a notebook with printed content (similar to PRISM) for 12 months.	Home- based	Older adults without cognitive impairments $\left(n=300\right)$	Social isolation, loneliness, perceived social support, and social network size (pretest, 6 months, posttest)	Friendship Scale, UCLA Loneliness Scale, Interpersonal Support Evaluation List, and Lubben Social Network Index	6 months: significant decrease in loneliness (b=1.72, p<0.04) and increase in perceived social support (b=−1.96, p<0.004) of the PRISM group vs. comparison group. 12 months: not maintained.
Matz-Costa et al. (2018) [71], USA	RCT	Engaged4Life program: (1) technology-assisted self-monitoring of physical activity for 8 weeks, (2) 3 hr psycho-education group session, (3) phone calls by peer mentors for 2.5 weeks (2×/week)	Comparison: technology-assisted self-monitoring of physical activity for 8 weeks.	Home- based	Older adults without cognitive impairments $\left(n=30\right)$	Social interaction (pretest within first week, week 4)	Survey related to the quantity and quality of social interaction	No significant changes in social interaction of the intervention group vs. comparison group.
Myhre, Mehl, and Glisky (2017) [73], USA	CCT	Facebook: 2 hr training sessions for 1 week (3×/week), use of Facebook (1×/day) and writing posts (1×/week) for 7 weeks	Comparison: online diary website. 2 hr training sessions for 1 week (3×/week), use of diary website (1×/day) and writing data entries (1×/week) for 7 weeks. Control: waitlist.	Home-based combined with training sessions at computer lab classrooms	Older adults without cognitive impairments $\left(n=43\right)$	Loneliness, social support, and social integration (pretest, posttest)	UCLA Loneliness Scale, Medical Outcomes Study Social Support Survey, Lubben Social Network Scale, and Social Provisions Scale	No significant differences in social support, loneliness, and social integration (pretest vs. posttest) in any of the groups.
Vanoh et al. (2019) [80], Malaysia	RCT	WESIHAT 2.0© (https://creativecommons.org/licenses/by-nc/3.0/, accessed on 14 April 2021) web-based wellness application: use of the application for 6 months, 30 min/day (4×/week) in combination with group counselling sessions in the first 3 months	Use of a health education pamphlet containing dietary recommendations for 6 months, in combination with dietary counselling.	Home-based combined with counselling sessions at a community hall	Older adults without cognitive impairment $\left(n=60\right)$	Loneliness and social support (pretest, 3 months, posttest)	Three-item loneliness scale and Medical Outcome Social Support Survey (MOSS)	Significant interaction effect for informational support $\left(\eta^{2}{}_{p}=0.123,\;p<0.05\right)$ and tangible support $\left(\eta^{2}{}_{p}=0.186,\;p<0.001\right)$. No statistically significant interaction effects for loneliness and other dimensions of social support.
Bickmore et al. (2005) [46], USA	CCT	Embodied Conversational Agent (ECA) “FitTrack”: daily interaction with the relational agent (who acted as an exercise advisor) for 2 months	Comparison: physical activity intervention for 2 months.	Home- based	Older adults without cognitive impairments $\left(n=21\right)$	Loneliness (pretest, posttest)	R-UCLA Loneliness Scale	Loneliness decreased statistically significant in the control group $\left(\mathrm{paired}\;t\left(7\right)=2.74,\;p<0.05\right)$, not in the intervention group. No significant group differences.
Woodward et al. (2011) [56], USA	CCT	Computer/Internet training program: 11 training sessions in a group delivered by the project coordinator for 6 months (1×/2weeks)	Control: no intervention.	Computer lab	Older adults $\left(n=83\right)$	Social support and loneliness (pretest, 3 months, posttest, 3 months following the training)	Self-reported social network data, Multidimensional Scale of Perceived Social Support (MSPSS), De Jong Gierveld Loneliness Scale	No statistically significant differences in social support and loneliness between the groups. Trend of higher perceived social support in intervention group vs. control group.
Kahlbaugh et al. (2011) [52], USA	CCT	Playing Wii: 1 hr activity with an undergraduate student for 10 weeks (1×/week)	Comparison: 1 hr watching television with an undergraduate student for 10 weeks (1×/week). Control: no visit.	Home- based	Older adults $\left(n=36\right)$	Loneliness (pretest, posttest)	UCLA Loneliness Scale	Significant decrease in loneliness from pretest to posttest in intervention group $\left(F\left(2,\;30\right)=6.24,\;p<0.005\right)$, increase in loneliness in comparison group.
Jansen- Kosterink et al. (2020) [67], the Netherlands	Cohort	Mobile application “GezelschApp” that stimulates users to engage in local activities together with other users: use of the application for 3 months combined with tailor-made coaching by a social worker	NA ^4^	Home- based	Older adults $\left(n=41\right)$	Loneliness (pretest, posttest)	De Jong Gierveld Loneliness Scale	Loneliness decreased among study participants (pretest vs. posttest). Not statistically significant.
Neil-Sztramko et al. (2020) [74], Canada	Cohort	iPad training program “AGE-ON”: 2 h education sessions (1×/week) for 6 weeks and use of the iPad/Internet at home	NA	Home-based (setting of training sessions not mentioned)	Older adults $\left(n=32\right)$	Social isolation and loneliness (pretest, posttest), social support (pretest, posttest, 1 month following the program)	Duke Social Support Index (DSSI), De Jong Gierveld Loneliness Scale, Lubben Social Network Scale	No significant differences in any social outcome measures.
Goumopoulos, Papa, and Stavrianos (2017) [65], Greece	One group mid- and posttest	Tablet-based intervention “Senior App Suite”: use of the mobile application suite for 8 weeks	NA	Home- based	Older adults without cognitive impairments $\left(n=22\right)$	Loneliness (pretest, posttest)	R-UCLA Loneliness Scale	“Senior App Suite” may reduce loneliness moderately $\left(p=0.034\right)$.

^1^ Study design as classified by the quality assessment (EPHPP). ^2^ RCT = randomized controlled trial. ^3^ CCT = clinical controlled trial. ^4^ NA = not applicable.

**Table 6:** Methods and main findings reported from included qualitative studies.

Authors (Year), Country	Technological Intervention	Setting	Participants (n= Sample Size)	Data Collection Methods	Main Findings
Hemberg and Fischer (2018) [66], Finland	Real video communication “CaringTV”	Home-based	Older adults $\left(n=7\right)$	Interviews	Overarching theme: “Being in a movement toward becoming a unity as human being” [66] (p. 93). Technology facilitated making new experiences, dedicating new meaning to everyday life, and maintaining or developing social contacts/relationships. Welfare technology as: “a window toward the world” [66] (p. 93).
Unbehaun et al. (2018) [76], Germany	Exergames program: regular use of the system combined with visits of trained research assistants 2×/week for 8 months	3 domestic environments and 4 day-care centers	Older adults with dementia n=14 and caregivers $\left(n=9\right)$	Semi-structured interviews and ongoing evaluation of the prototype	Benefits for people with dementia: enhanced physical skills, increased motivation, showed learning effects, increased social interaction and sense of interpersonal relationships (in day-care home setting), improved daily life routine. Benefits for caregivers: relief for caregivers (e.g., freeing up time).
Chi et al. (2017) [61], USA	Digital pet avatar: daily interaction with a conversational agent (a cat or dog avatar) for 3 months	Home-based	Older adults without cognitive impairment $\left(n=10\right)$	Secondary analysis of semi-structured interviews	Benefits: provided companionship, reminders, a journal, entertainment, increased social interaction and physical activity. System challenges: technical issues and the limited ability to make conversations. Major concerns: privacy, costs, and dependence.
Ballantyne et al. (2010) [45], Australia	Internet Social Networking Website (ISNW) “About My Age”: one-on-one education sessions delivered by project team members for 3 months (in the beginning, weekly support visits, then less frequently)	Home-based	Older adults $\left(n=6\right)$	Semi-structured interviews and reflective journals of the project team	Benefits: enabled exploration of other ways of communication, contributed to a positive and personalised learning experience (using the one-on-one approach), reduced temporal loneliness (extent varied per case), increased sense of connectivity to the outside world to some extent.
Biniok and Menke (2015) [47], Germany	Tablet with communication platform “SONIA”: training sessions in groups delivered by researchers and volunteers and use of the platform for 6 months	Home-based combined with training sessions at a university/community college	Older adults $\left(n=30\right)$	Group discussions and observations	ICT created, extended, and facilitated engagement in participation space: Participants with few social contacts: enhanced technological skills, increased self-esteem, and increased social participation. Socially active participants: growth and intensification of social contacts/interactions. Some participants (mostly with high technological skills): only slight changes in social participation.
Bowes and McColgan (2012) [48], UK	Telecare	Home-based	Older adults $\left(n=76\right)$ and family caregivers $\left(n=16\right)$	Semi-structured interviews	Independence: promoted participants’ confidence, feelings of safety, and freedom. Social Participation: enabled living in the community, enhanced relationships, but led to restriction in activities formerly enjoyed and narrowing of social networks. Identity: contributed to a positive sense of identity.
Breck, Dennis, and Leedahl (2018) [59], USA	Cyber-Seniors Program: technology training lessons delivered by young adult mentors using reverse mentoring 1×/week	Senior center and other locations	Older adults $\left(n=29\right)$ and young adult mentors $\left(n=28\right)$	Session logs of young adult mentors and surveys	Benefits for older adults: gained confidence in the use of technology to make social connections digitally. Benefits for young adult mentors: enhanced leadership skills. Both: Age-related stereotypes were challenged. Intergenerational engagement and connections emerged.
Judges et al. (2017) [68], Canada	Digital communication tool “InTouch”: social contact using the system with a paired volunteer 1×/week for 3 months	Home-based	Older adults $\left(n=10\right)$ and volunteer participants $\left(n=10\right)$	Semi-structured interviews, field notes of volunteer participants and the study coordinator, and data logs	Benefits: improved communication and positive changes in relationships. Use of technology led to mixed feelings in study participants. Adoption: 4 of the study participants were able to adopt “InTouch”. Internal motivation contributed to successful adoption. Key barriers to adoption: lack of volunteer support, social difficulties, and diverse health issues.
Kim and Gray (2016) [69], USA	Computer training program: use of computer and 1 h training sessions of computer/Internet skills (1×/week)	Home-based combined with training sessions at senior housing facilities	Low-income older adults $\left(n=11\right)$	Semi-structured interviews and interviewer’s field notes	Benefits: enhanced social connections, monetary benefits, and development of life skills. Barriers to program participation: fear of technology, low literacy, and distrust of governmental programs. Barriers to sustained Internet use: problems and costs of broadband services, concerns about cyber security, and limited proficiency. Success factors to sustained Internet use: ongoing technical support and individual ICT devices.
O’Brien, Smith, and Beck (2016) [75], USA	3D virtual world “Second Life” (SL): training/onboarding for two weeks, SL events organized by trained staff for 8 weeks, independent use of SL for 2 weeks	Home-based	Older adults $\left(n=51\right)$	Semi-structured interviews	Older adults reported to be open to the possibility of creating online relationships within the virtual world. Most of the participants did not succeed in creating them. Obstacles to the formation of online relationships: personality, difficulties with other avatars, and lack of face-to-face interactions.
Cutler, Hicks, and Innes (2015) [62], UK	Digital gaming training program: 2 h training sessions (“Tech Clubs”) in groups delivered by facilitators for 6–8 weeks	Home-based combined with training sessions at 4 different venues	Older adults with dementia $\left(n=29\right)$	Ethnographic field notes, self-complete questionnaires, and focus groups	Impact of digital gaming on healthy aging: promoted lifelong learning; increased physical activity, social interaction, and mental stimulation; and promoted independence.
Airola, Rasi, and Outila (2020) [57], Finland	Phone and video conferencing (VC) service: calls from a volunteer 1×/week	Home-based	VC service coordinator, volunteers, and older adult service users $\left(n=5\right)$	Semi-structured interviews	Barriers to learning and using the service: technical problems, volunteer–user relationship, lack of technical skills, health status, and a negative attitude toward technology. Enablers to learning and using the service: technical support, social support networks, previous experience with technology, and a positive attitude toward new technologies. Benefits: facilitated to establish networks and reduce loneliness.
Cornejo, Tentori, and Favela (2013) [49], Mexico	Ambient Social Network System “Tlatoque”: use of an interactive display for 21 weeks	Home-based	Older adults $\left(n=2\right)$ and family members $\left(n=30\right)$	Semi-structured interviews and a focus group	Tlatoque supported social connectedness through: a higher frequency of social contacts (consensual meetings or opportunistic encounters around the system). the strengthening of social ties between the older adult and family members.
Burmeister et al. (2016) [60], USA	iPad training program: 2 h training sessions in groups delivered by a peer trainer 1×/week for 4 months	Home-based combined with training sessions at a Seniors Citizen’s Club	Older adults $\left(n=6\right)$	Interviews, participants’ diaries, researchers’ observations, and peer trainer’s reports	Benefits: enhanced ICT skills, increased social connectedness, and improved life satisfaction. Important factors: individualized education approach and social connections between participants and peer trainer.

**Table 7:** Design, methods, and findings related to social participation reported from included mixed methods studies.

Authors (Year), Country	Experimental Intervention	Setting	Participants ( n= Sample Size)	Outcomes Related to Social Participation	Quantitative Outcome Measures Related to Social Participation	Qualitative Data Collection Methods	Findings Related to Social Participation
Hind et al. (2014) [51], UK	One-on-one telephone friendship (TF) vs. usual care control: (1) 10 to 20 min calls delivered by volunteer facilitators for six weeks (1×/week), followed by (2) 1 h TF groups for 12 weeks (1×/week)	Home-based	Older adults without cognitive impairments $\left(n=157\right)$	Loneliness (pretest, 6 months follow-up post randomization)	De Jong Gierveld Loneliness Scale	Semi-structured interviews	Loneliness: no statistically significant improvement. Interviews: participant reported a lack of face-to-face contact and a dissatisfaction with group cohesion.
Ware et al. (2017) [77], France	Language training program: 2 h sessions of an English language training delivered by a native English-speaking psychologist using a tablet-based multimedia approach for 4 months (1×/week)	Laboratory of a hospital	Older adults without cognitive impairments $\left(n=14\right)$	Loneliness (pretest, posttest)	UCLA Loneliness Scale, and semi-structured interviews	Semi-structured interviews	Loneliness: no statistically significant improvement. Interviews: participants reported that they did not build strong social ties with other group participants.
Lee and Kim (2018) [70], USA	Intergenerational Mentor-Up (IMU) class: six 1 h one-on-one technology tutorial sessions delivered by college students (partly in groups)	Senior centers and housing facilities	Older adults without cognitive impairments $\left(n=59\right)$	Social isolation (pretest, posttest)	Perceived social isolation measure (loneliness and social support) and self-reported life stressors checklist	Interviews and researchers’ field notes	Total social isolation significantly decreased (t=3.84, p<0.001,d=0.74), with no significant change in lack of social support and a statistically significant decrease in loneliness (t=7.53, p<0.001,d=1.45).
Mullins et al. (2020) [72], USA	Internet Information Station program: three different computer classes delivered by students	Four apartment buildings of a Housing and Urban Development community	Older adults participating in program $\left(n=262\right)$ Older adults filling in the R-UCLA Loneliness Scale $\left(n=11\right)$	Loneliness (pretest, posttest at 4–6 weeks after the program)	R-UCLA Loneliness Scale	Ethnographic interviews and observations	Participants reported enhanced social connectedness. Observed increase in participation in the common areas of the Housing and Urban Development community. Decrease in loneliness of the technology class group (vs. baseline group): significant change $\left(p=0.023\right)$ on the item “There is no one I can turn to”.
Engelbrecht a nd Shoemark (2015) [50], Australia	Activity-based musical engagement using iPads vs. Traditional Music Instruments (TMI): 1 h sessions of activity-based musical engagement in groups delivered by a therapist for 5 weeks (1×/week)	Not mentioned	Older adults without cognitive impairments $\left(n=6\right)$	Social isolation (pretest, posttest)	Friendship scale	Journal entries, researcher’s field notes, and session reflections	No significant differences in social isolation (1) between the iPad and the TMI group and (2) within the groups (pre- vs. posttest). Reported benefits for both groups: enhanced positive self-concepts and developed social cohesion and group identity.
Zaine et al. (2019) [79], UK and Brazil	Human-facilitated social networking system “Media Parcels”: use of the tablet-based system facilitated by a clinical psychologist for two weeks with family members (trial 1) or friends (trial 2)	Home-based	Older adults $\left(n=2\right)$, family members $\left(n=2\right)$, and older adult friends $\left(n=2\right)$	Feelings of social connection (pretest, week 1, posttest)	Self-developed Relationship Semantic Differential Scale (RSDS)	Interviews	Participants reported contacting each other more often and feeling closer to each other.
Arthanat, Vroman, and Lysack (2016) [58], USA	iPad training program: individualized one-on-one training sessions delivered by a coach (occupational therapy student) for 3 months (1×/month), then iPad use without assistance for 3 months	Home-based	Older adults without cognitive impairments $\left(n=13\right)$	Breadth and frequency of technology use related to social connections (pretest, 1 month, 2 months, 3 months, 4 months, posttest)	Self-developed questionnaire ^1^	Field observations, self-developed end-of-study questionnaire, and focus groups	Modest (not significant) increase in activities involving social connections. Participants identified benefits and challenges of the program related to technology experiences, interactions with the coach, the training approach, and specific activities.
Mellor, Firth, and Moore (2008) [53], Australia	Providing internet access: use of computer/Internet for 12 months (with support on a daily basis for the first two weeks)	Retirement villages	Older adults $\left(n=20\right)$	Social connectedness (pretest, 3 months, 6 months, 9 months, posttest)	Social Connectedness Scale	Semi-structured interviews	At 12 months: no significant differences in social connectedness. Benefits reported in interviews: positive impact on social connectedness.
Ring et al. (2015) [54], USA	ECA ^2^ motion sensor vs. non-sensor condition: interact with the ECA on a touchscreen computer (1×/day) for 1 week	Home-based	Older adults $\left(n=14\right)$	Loneliness (pretest, posttest)	UCLA Loneliness Scale	Diary entries and semi-structured interviews	Significant lower loneliness in intervention group vs. comparison group when interacting with the ECA $\left(F\left(1,150\right)=7.713,\;p<0.01\right)$. This indicates that the ECA is more effective in reducing loneliness when using a motion sensor to actively initiate social interactions with older adults.
Emas et al. (2018) [64], USA	iPad/iPhone training program: 1 h multimodal training sessions in groups 1×/week for 7 weeks	Home-based combined with training sessions at a gated retirement community	Older adults $\left(n=25\right)$	Participants’ skill ability (PSA); participants’ confidence level (PCL) (pretest, posttest)	Self-developed scales measuring PSA and PCL	Journaling prompts	Statistically significant increase in PSA of defining several Internet acronyms and statistically significant increase in PCL using FaceTime $\left(t\left(16\right)=6.85,\;p=0.00\right)$, and taking photos $\left(t\left(16\right)=4.26,\;p=0.0001\right)$. Facilitation of social participation: participants reported to have gained skills and knowledge in communicating with loved ones using concepts such as FaceTime, texts, e-mails, and phone calls.

^1^ Only two of the four categories of ICT activities were relevant: family connections and social connections. ^2^ ECA = embodied conversational agent.

## Supplementary Materials

The following are available online at https://www.mdpi.com/article/10.3390/jcm10112308/s1, Tables S1–S6: Specific survey see in supplementary materials.
