# Supplementary material for: The Effects of Technological Interventions on Social Participation of Community-Dwelling Older Adults with and without Dementia: A Systematic Review
Source: J Clin Med. 2021 May 25;10(11):2308. doi: 10.3390/jcm10112308 (PMC8198527; doi:10.3390/jcm10112308)
Supplement: Supplementary file 1 [file jcm-10-02308-s001.zip › Appendix A.pdf]

# Appendix A: Screening Tool

**Citation and Title Screening**

1. Does the citation indicate publication on or after 2000?

Yes: continue screening
No: stop screening

2. Does the title use English?

Yes: continue screening
No: stop screening

3. Is an abstract available?

Yes: continue screening
No: stop screening

**Abstract/Full-text Study Screening**

4. Does the abstract/full-text study use English?

Yes: continue screening
No: stop screening

5. Does the abstract/full-text study indicate that the study evaluated an intervention (an intervention is tested and effects are described – doesn't matter if qualitative, quantitative, or mixed-methods design)?

Yes or Unsure: continue screening
No: stop screening

6. Does the abstract/full-text study indicate that the studied intervention used technology?

Yes or Unsure: continue screening
No: stop screening

7. Does the abstract/full-text study indicate that the study population is aged 55 and older?

Yes: continue screening
No: stop screening

8. Does the abstract/full-text study indicate that the study population consist of healthy older adults/older adults in general OR older adults with cognitive impairments or dementia?

Yes: continue screening
*Definition cognitive impairments/dementia: people with a diagnosis of Alzheimer's disease, vascular dementia, frontotemporal dementia, Pick's disease, Lewy body dementia, Korsakoff syndrome, Creutzfeldt-Jakob disease, Parkinson's disease dementia, Posterior Cortical Atrophy (PCA), cognitive impairment – such as Mild Cognitive Impairment (MCI) – and non-specified dementia*
No: stop screening

9. Does the abstract/full-text study indicate that the older adults were community-dwelling?

Yes or Unsure or not mentioned: continue screening
*For example: the study population was living independently in the community, living in the community with friends, family and/or informal caregivers, or living in an assisted living facility/residential aged care.*
No: stop screening
*For example: The study population was living in a nursing home.*

10. Does the abstract/full-text study indicate that the at least one outcome is related to the older adult with or without cognitive impairment or dementia)?

Yes or Unsure: continue screening

No: stop screening
*For example: The outcomes are related to the intervention (e.g. the acceptability/usability of the intervention/technology).*

11. (a) Does the abstract/full-text study clearly state that the intervention was intended to improve social participation or to reduce social isolation/loneliness of the older adult?

OR

(b) Does the abstract/full-text study indicate that at least one outcome is related to the social participation/social isolation/loneliness of the older adult?
Yes or Unsure or not mentioned: continue screening
*We use the definition of social participation by Levasseur, Richard, Gauvin, and Raymond: a "person's involvement in activities that provide interaction with others in society or the community". (2010, p. 2148)*[1]
No: stop screening

**Inclusion/Exclusion Decision**

a. Included, all questions were answered "Yes", "Unsure" or "Not mentioned"
b. Excluded, at least one question was answered definitely "No"

[1] Levasseur, M.; Richard, L.; Gauvin, L.; Raymond, É. Inventory and analysis of definitions of social participation found in the aging literature: Proposed taxonomy of social activities. *Soc. Sci. Med.* **2010**, *71*, 2141-2149. doi:10.1016/j.socscimed.2010.09.041
