# Supplementary material for: The Effects of Technological Interventions on Social Participation of Community-Dwelling Older Adults with and without Dementia: A Systematic Review
Source: J Clin Med. 2021 May 25;10(11):2308. doi: 10.3390/jcm10112308 (PMC8198527; doi:10.3390/jcm10112308)
Supplement: Supplementary file 1 [file jcm-10-02308-s001.zip › Appendix B.pdf]

Table S1: Data collection form: technological interventions to enhance social participation in dementia

Version 2, February 2021

Study title	
Study ID *(surname of first author and year first full report of study was published e.g. Smith 2010)*	
Paper ID *(from Excel file)*	
Date form completed *(dd/mm/yyyy)*	
Publication type *(e.g. full report, paper, conference paper)*	

Table S2: General Study Characteristics

	**Descriptions as stated in report/paper**	**Location in text or source** *(pg. & ¶/fig/table/other)*
**Aim of study** *(e.g. efficacy, equivalence, pragmatic)*		
**Design of study** *(please specify how the authors define the design)*		
**Country of data collection** *(please specify location and the social setting)*		
**Ethical approval obtained for study**	☐ Yes ☐ No ☐ Unclear/not described	

**Theoretical concepts related to social participation** *(e.g. social connectedness, social isolation, loneliness, … specify the definition used)*		
**Study population**	__ Older adults in general (excluding older adults with cognitive impairments)<br>__ Older adults in general (including older adults with cognitive impairments)<br>__ Older adults with cognitive impairments<br>Other, namely________________________________	
	**Participants**<br>*N:*<br>*Mean age:*<br>*Age range:*<br>*Male:*<br>*Female:*	
	**Inclusion/exclusion criteria**	
	**Withdrawals, exclusions and drop-outs** *(provide reasons if stated)*	

Table S3: Intervention characteristics

	**Description as stated in report/paper**	**Location in text or source** *(pg. & ¶/fig/table/other)*
**Description of the intervention/ technology**	**Experimental intervention: (N =)**<br><br>**Comparison intervention: (N =)**	
**Aim of intervention**		
**Duration of intervention period**		
**Timing** *(e.g. frequency, duration of each episode)*		
**Providers** *(e.g. research assistant, health care professional, researcher)*		
**Setting**		
**Theoretical basis of the intervention**		

Table S4: Outcomes

Copy and paste table for each outcome

	**Description as stated in report/paper**				**Location in text or source** *(pg. & ¶/fig/table/other)*
**Outcome name** *(just for quantitative/mixed methods studies)*					
**Outcome definition** *(just for quantitative/mixed methods studies)*					
**Time points measured** *(specify whether from start or end of intervention)*					
**Outcome measures** *(please tick and specify the data collection methods)*	☐ *QUANTITATIVE*	☐ *QUALITATIVE*	☐ *MIXED METHODS*		
**Scales: upper and lower limits** *(indicate whether high or low score is good, just for quantitative/mixed methods studies)*					
**Is outcome/tool validated?** *(just for quantitative/mixed methods studies)*	☐ Yes	☐ No	☐ Unclear	☐ Not applicable	

Table S5: Findings and influencing factors

	Description as stated in report/paper	Location in text or source *(pg. & ¶/fig/table/other)*
**Main findings of the study**		
**Factors explaining the success or failure of the intervention in influencing social participation**		

Table S6: Other information

**Key conclusions of study authors**	
**Correspondence for further study information**	
**Notes:**	
